# Cardiac Fibroblast Growth Factor 23 Excess Does Not Induce Left Ventricular Hypertrophy in Healthy Mice

**DOI:** 10.3389/fcell.2021.745892

**Published:** 2021-10-28

**Authors:** Maren Leifheit-Nestler, Miriam A. Wagner, Beatrice Richter, Corinna Piepert, Fiona Eitner, Ineke Böckmann, Isabel Vogt, Andrea Grund, Susanne S. Hille, Ariana Foinquinos, Karina Zimmer, Thomas Thum, Oliver J. Müller, Dieter Haffner

**Affiliations:** ^1^Department of Pediatric Kidney, Liver and Metabolic Diseases, Pediatric Research Center, Hannover Medical School, Hanover, Germany; ^2^Department of Internal Medicine III, University Hospital Kiel, Kiel, Germany; ^3^German Center for Cardiovascular Research (DZHK), Partner Site Hamburg/Kiel/Lübeck, Kiel, Germany; ^4^Institute of Molecular and Translational Therapeutic Strategies, Hannover Medical School, Hanover, Germany; ^5^National Heart and Lung Institute, Imperial College London, London, United Kingdom; ^6^REBIRTH Center for Translational Regenerative Medicine, Hannover Medical School, Hanover, Germany

**Keywords:** FGF23, klotho, left ventricular hypertrophy, fibrosis, mineral metabolism, mouse model

## Abstract

Fibroblast growth factor (FGF) 23 is elevated in chronic kidney disease (CKD) to maintain phosphate homeostasis. FGF23 is associated with left ventricular hypertrophy (LVH) in CKD and induces LVH *via* klotho-independent FGFR4-mediated activation of calcineurin/nuclear factor of activated T cells (NFAT) signaling in animal models, displaying systemic alterations possibly contributing to heart injury. Whether elevated FGF23 *per se* causes LVH in healthy animals is unknown. By generating a mouse model with high intra-cardiac Fgf23 synthesis using an adeno-associated virus (AAV) expressing murine Fgf23 (AAV-Fgf23) under the control of the cardiac troponin T promoter, we investigated how cardiac Fgf23 affects cardiac remodeling and function in C57BL/6 wild-type mice. We report that AAV-Fgf23 mice showed increased cardiac-specific *Fgf23* mRNA expression and synthesis of full-length intact Fgf23 (iFgf23) protein. Circulating total and iFgf23 levels were significantly elevated in AAV-Fgf23 mice compared to controls with no difference in bone *Fgf23* expression, suggesting a cardiac origin. Serum of AAV-Fgf23 mice stimulated hypertrophic growth of neonatal rat ventricular myocytes (NRVM) and induced pro-hypertrophic NFAT target genes in klotho-free culture conditions *in vitro*. Further analysis revealed that renal Fgfr1/klotho/extracellular signal-regulated kinases 1/2 signaling was activated in AAV-Fgf23 mice, resulting in downregulation of sodium-phosphate cotransporter *NaPi2a* and *NaPi2c* and suppression of *Cyp27b1*, further supporting the bioactivity of cardiac-derived iFgf23. Of interest, no LVH, LV fibrosis, or impaired cardiac function was observed in klotho sufficient AAV-Fgf23 mice. Verified in NRVM, we show that co-stimulation with soluble klotho prevented Fgf23-induced cellular hypertrophy, supporting the hypothesis that high cardiac Fgf23 does not act cardiotoxic in the presence of its physiological cofactor klotho. In conclusion, chronic exposure to elevated cardiac iFgf23 does not induce LVH in healthy mice, suggesting that Fgf23 excess *per se* does not tackle the heart.

## Introduction

Circulating levels of the bone-derived phosphaturic hormone fibroblast growth factor (FGF) 23 rise progressively as kidney function declines to maintain phosphate homeostasis. FGF23 enhances renal phosphate excretion *via* FGF receptor (FGFR) 1/klotho-mediated activation of extracellular signal-regulated kinases (ERK) 1/2 signaling resulting in downregulation of sodium-phosphate cotransporters *NaPi2a* and *NaPi2c* (Isakova et al., 2011). In addition, FGF23 suppresses renal 1,25(OH)_2_D_3_ (1,25OHD) synthesis by inhibiting 1α-hydroxylase (CYP27B1) and stimulating 24-hydroxylase (CYP24A1) resulting in decreased intestinal phosphate absorption (Musgrove and Wolf, 2020).

Fibroblast growth factor 23 exists in an intact, biologically active protein (iFGF23) that is stabilized by O-glycosylation *via* polypeptide *N*-acetygalactosaminyltransferase 3 (GALNT3) at threonine 178. Cleavage of FGF23 by furin due to the family with sequence similarity 20, member C (Fam20C)-mediated phosphorylation of serine 180 at the cleavage site results in C- and N-terminal fragments (Tagliabracci et al., 2014). iFGF23 induces hypertrophic growth of cardiac myocytes *in vitro* and left ventricular hypertrophy (LVH) in rodents *via* FGFR4-mediated activation of the calcineurin/nuclear factor of activated T cells (NFAT) signaling pathway independent of its physiological cofactor klotho, indicating that FGF23 can directly affect the heart (Faul et al., 2011; Grabner et al., 2015). FGF23 expression is not limited to the bone, as cardiac myocytes express FGF23, too. Recent studies demonstrate that its expression is increased in cardiac and kidney injury (Andrukhova et al., 2015; Richter et al., 2015; Leifheit-Nestler et al., 2016, 2017), suggesting that cardiac toxicity of FGF23 may be at least partly due to the paracrine effects of heart-derived FGF23. Several studies questioned whether elevated FGF23 *per se* is able to induce pathologic alterations in the heart (Slavic et al., 2017; Leifheit-Nestler et al., 2018b; Liu et al., 2018; Pastor-Arroyo et al., 2018; Zhou et al., 2018). Likewise, a meta-analysis evaluating the association between FGF23 and cardiovascular outcomes found no significant exposure-response relationship in patients with and without chronic kidney disease (CKD) (Marthi et al., 2018).

Here, we generated a mouse model with cardiac-specific overexpression of Fgf23 *via* myocardial gene transfer using adeno-associated virus (AAV) in order to elucidate pathological properties of elevated cardiac Fgf23 in unchallenged healthy wild-type mice. Using isolated neonatal rat ventricular myocytes (NRVM), *in vivo* data were verified and cardioprotective mechanisms were studied.

## Materials and Methods

### Adeno-Associated Virus Vector Production

The murine full-length wild-type *Fgf23* cDNA was PCR amplified and inserted into a double-stranded AAV genome plasmid (scAAV-TnT-EGFP) under the control of the cardiac troponin T promoter after excision of EGFR (Werfel et al., 2014) generating a scAAV-TnT-Fgf23 (AAV-Fgf23) vector genome. AAV serotype 9 vectors were produced, purified, and titrated by qPCR on vector genomes as previously described (Jungmann et al., 2017).

### Animal Experiments

For the pilot studies, 8-week-old male C57BL/6N mice (Charles River) were injected s.c. with 2 × 10^9^ vector genomic particles (vg), 5 × 10^11^ and 10^12^ vg AAV-Fgf23, respectively, or AAV-vehicle as control (Ctrl) and cardiac magnetic resonance imaging (MRI) as well as cardiac-specific induction of Fgf23 were analyzed at different time points as indicated. For the main study, 25 8-week-old male C57BL/6N mice were randomly assigned into two groups. Fifteen mice were injected s.c. with 5 × 10^11^ vg AAV-Fgf23 and 10 mice received the Ctrl. Mice were fed normal rodent chow (#1324, Altromin, Germany) *ad lib* for 4 months followed by MRI and echocardiography. Spontaneous urine was collected and whole blood was sampled by cardiac puncture. Femur, tibia, and kidneys were isolated and snap-frozen in liquid nitrogen. Isolated mouse hearts were washed in PBS followed by 0.5% potassium chloride and cut into three cross-sections. For histological evaluation, the mid cross-section was fixated in 4% RotiHistofix (Carl Roth) and embedded in paraffin. The other two cross-sections were snap-frozen in liquid nitrogen and stored at −80°C for molecular and biochemical analyses. Serum and urine samples were quantified for phosphate, calcium, and creatinine (all Abcam). ELISA-based analyses were performed for murine C-term FGF23, intact FGF23 (iFgf23), and PTH1-84 (all Immutopics) using plasma samples and whole heart tissue lysates. Tubular reabsorption of phosphate (TRP) was calculated as described elsewhere (Barth et al., 2000).

### Magnetic Resonance Imaging

MRI imaging was performed using a 7T Bruker PharmaScan 70/16 US (Bruker BioSpin GmbH), equipped with a four-element cardiac coil array along with a linear volume resonator. Mice were anesthetized with 3% isoflurane. The heart was displayed in short-axis images using a multi-slice FLASH cine sequence. MRI images were analyzed with Mass4Mice (Medis Medical Imaging Systems). Epicardium and endocardium were marked manually to ascertain left ventricular (LV) mass, end-systolic (ES) and end-diastolic (ED) volume, stroke volume (SV), cardiac output, and ejection fraction (EF). LV wall geometry was determined at the level of papillary muscles. With regard to slice thickness, papillary muscles were included in the LV cavity.

### Echocardiography

Echocardiography was performed using Vevo 2100 (FUJIFILM Visual Sonics). Mice were anesthetized with 3% isoflurane, monitoring heart rate to above 400 bpm for comparable results between groups. PLAX view in B and M-Mode images and four-chamber view were taken and analyzed for heart function and geometry using Vevo Lab Software (Visual Sonics). Heart function and geometry were assessed including EF, SV, cardiac output, fractional shortening (FS), LV anterior and posterior wall thickness (LVAW and LVPW), LV diameter (LVID), and LV volumes.

### Cell Culture

Neonatal rat ventricular myocytes were isolated from Sprague–Dawley rats by Percoll gradient centrifugation and cultured as previously described (Böckmann et al., 2019). After starvation in 20% M199 overnight, NRVM were stimulated for 48 h with either vehicle, 20 μM phenylephrine (PE), 20 ng/mL recombinant oncostatin M (OSM; Ala24-Arg206) in the absence or presence of 100 ng/mL recombinant soluble αKlotho [sKL; 6 His-tagged Ala35-Lys982 (Arg948Lys)] (all R&D Systems), or 2% serum from Ctrl and AAV-Fgf23 mice, respectively. To analyze hypertrophic cell growth, NRVM were fixed in 4% paraformaldehyde, permeabilized in Triton X-100, and incubated with mouse sarcomeric α-actinin antibody (EA-53, Sigma Aldrich) followed by incubation with goat anti-mouse secondary antibody. 4′,6-Diamidino-2-phenylindole (DAPI, Sigma Aldrich) was used to visualize nuclei. Immunofluorescence images were taken on a Zeiss Axio Observer Z1 microscope (Carl Zeiss) with a 20× objective. Cardiac myocyte cross-sectional area was quantified in at least 100 cells per group using Carl Zeiss ZEN software.

### RNA Isolation, Quantitative Real-Time PCR, and Semi-Quantitative PCR

RNA isolation from bone was done by RNeasy Lipid Tissue Mini Kit and all other RNA isolations were performed using RNeasy Mini Kit according to the respective manufacturer’s protocol. Transcription of 500 ng or 1 μg (bone) total RNA into cDNA was done with QuantiTect Reverse Transcription Kit and QuantiFast SYBR Green PCR Kit including Rox dye (Qiagen) was used to analyze gene expression. Detection was performed in triplicate using 17900HT Fast Real-Time PCR system and analyzed with SDS 2.4 Software (Applied Biosystems). For primer sequences see Supplementary Tables 1, 2. Relative mRNA expression in AAV-Fgf23 mice or NRVM was calculated according to 2^–ΔΔCt^ method using *Gapdh* (for heart, kidney, and NRVM) and *Rsp18* (for bone) as housekeeping genes, respectively.

For semi-quantitative PCR analysis of AAV, templates were mixed with 10× Coral Load PCR Buffer, 25 mM MgCl, 10 mM dNTPs, 5 U/μL Taq DNA Polymerase, RNase free water (Qiagen), and 1 mM primer mix (Supplementary Table 3). PCR was performed in duplicate with 45 cycles (94°C, 30 s; 60°C, 30 s; and 72°C, 1 min) and samples were separated using 2% agarose gel. Images were taken with BioRad Molecular Imager Gel Doc XRt and analyzed with Image Lab Software.

### Protein Isolation and Immunoblotting

For protein isolation, 10 mg heart tissue was homogenized with Qiagen TissueLyser and lysated in RIPA buffer including proteinase and phosphatase inhibitors (Sigma-Aldrich). One hundred micrograms total protein was separated on SDS-PAGE and transferred to nitrocellulose membrane. After blocking, primary antibodies (Supplementary Table 4) were incubated overnight followed by incubation of HRP or fluorescent-labeled secondary antibodies (Supplementary Table 5). Protein detection was performed using Odyssey Imager and analyzed with Image Studio 5.2 (LI-COR Biosciences).

### Histology

Three micrometers thick cardiac mid-chamber sections were deparaffinized and hematoxylin/eosin (HE) staining was performed using a standard protocol. To determine the cross-sectional area of individual cardiac myocytes, sections were incubated with wheat germ agglutinin (WGA) Alexa Fluor 555 (Invitrogen) and DAPI. Five pictures of the LV were taken using Zeiss Axio Observer Z1 microscope with a 40× magnification and at least 100 cells per mouse were analyzed with Carl Zeiss Zen software. For the quantification of interstitial LV fibrosis, sections were incubated using picrosirius red solution (Merck) in 1.2% picric acid and analyzed using ImageJ. Immunofluorescent staining with primary anti-Fgf23 antibody (dilution 1:200; Bioss #bs-5768R) followed by incubation with goat-anti-rabbit Alexa Fluor 555 secondary antibody (dilution 1:500; Invitrogen #A21428) was performed to verify the localization of AAV-Fgf23 in cardiac myocytes.

### Statistical Analysis

Statistical analysis was performed using GraphPad Prism software version 9. ROUT method with a Q of 0.1% was performed to identify outliers. Gaussian distributed data according to the D’Agostino and Pearson (*n* ≥ 8) or Shapiro–Wilk (*n* < 8) normality test was analyzed using the two-tailed *t*-test, while for non-Gaussian distributed data the Mann–Whitney test was applied. For analysis of more than two groups, one-way ANOVA or Kruskal–Wallis tests followed by Sidak’s or Dunn’s multiple comparisons *post hoc* tests were performed. Significance was presumed when *p* < 0.05.

## Results

### Evaluation of Time and Dose-Dependent AAV-Fgf23 Administration

**FIGURE 1:**
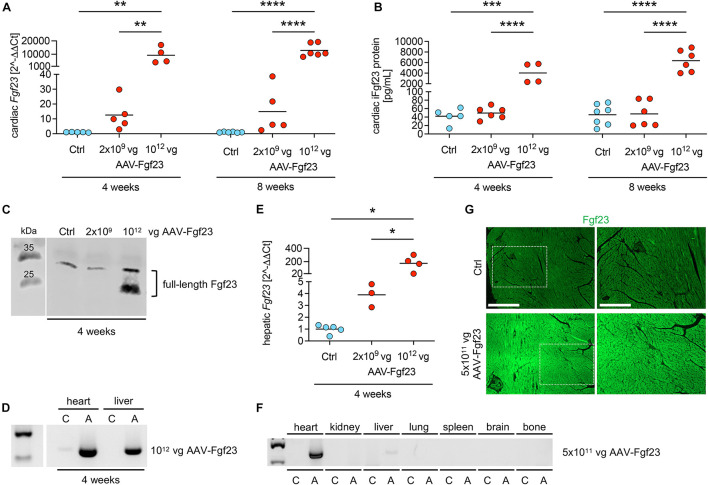
Dose- and time-dependent evaluation of AAV-Fgf23. **(A)** Relative cardiac *Fgf23* mRNA expression is dose-dependently increased in AAV-Fgf23 mice compared to Ctrl, irrespective of the duration of exposure. **(B)** ELISA-based quantification of cardiac Fgf23 protein in heart tissue lysates shows increased intact Fgf23 (iFgf23) protein only in AAV-Fgf23 mice administered with 10^12^ vector genomic particles (vg) compared to Ctrl. **(C)** Representative immunoblotting of Fgf23 in cardiac tissue demonstrates increased iFgf23 protein in the 10^12^ vg AAV-Fgf23 group after 4 weeks compared to Ctrl. **(D)** Semi-quantitative PCR targeting AAV vector shows excessive virus transduction after 4 weeks in the heart and the liver of 10^12^ vg AAV-Fgf23 mice (A) compared to Ctrl (C). **(E)** Hepatic *Fgf23* mRNA expression is significantly increased after 4 weeks in 10^12^ vg AAV-Fgf23 mice compared to Ctrl. **(F)** By injecting 5 × 10^11^ vg AAV-Fgf23, semi-quantitative PCR targeting AAV vector shows distinct virus transduction in the heart compared to Ctrl, whereas transduction in liver tissue was very low and absent in kidney, lung, spleen, brain, and bone tissue. **(G)** Immunofluorescent staining of cardiac mid-chamber sections demonstrates uniform Fgf23 (green) overexpression in almost all cardiac myocytes of the left ventricle in AAV-Fgf23 mice compared to Ctrl (original magnification ×20; scale bar, 500 and 250 μm, respectively). Data are given as scatter dot plots with means; *p < 0.05, **p < 0.01, ***p < 0.001, and ****p < 0.0001 analyzed using one-way ANOVA or Kruskal–Wallis test followed by Sidak’s or Dunn’s multiple comparisons *post hoc* tests, respectively, according to Shapiro–Wilk normality test; *n* = 3–7 mice per group.

First, we evaluated the time- and dose-dependent effects of AAV-Fgf23-mediated cardiac-specific overexpression of Fgf23 in mice using the AAV serotype 9, reported as the most efficient serotype for cardiac gene transfer in rodents, with the cardiac-specific troponin T promoter for increased heart tropism (Inagaki et al., 2006; Merentie et al., 2016). Injection of AAV-Fgf23 resulted in a dose-dependent induction of cardiac *Fgf23* mRNA expression that reached statistical significance only when using the highest dose of 10^12^ vg AAV-Fgf23, independent of the post-injection time (Figure 1A). ELISA-based quantification in whole heart tissue lysates and western blot analysis revealed a higher synthesis of iFgf23 protein only in the 10^12^ vg AAV-Fgf23 group (Figures 1B,C). Although, 10^12^ vg AAV-Fgf23 caused an accumulation of AAV in the heart after 4 weeks, an unspecific detection of AAV in the liver with concomitant enhanced mRNA expression of hepatic *Fgf23* was observed as well (Figures 1D,E). Such off-target effects in the liver were previously reported comparing organ specificity of different AAV serotype 9 doses (Konkalmatt et al., 2013). Halving the AAV-Fgf23 concentration to 5 × 10^11^ vg specifically enhanced AAV expression in the heart compared to Ctrl with minimal accumulation in the liver and no transduction in kidney, lung, spleen, brain, or bone tissue of AAV-Fgf23 mice (Figure 1F). Important to note, delivery of 10^12^ vg AAV-Fgf23 caused an increase of *Fgf23* in the heart even after 4 weeks, which was equal to the amount of cardiac *Fgf23* in AAV-Fgf23 mice after 8 weeks. Injection of 5 × 10^11^ vg AAV-Fgf23 also still showed a high overexpression of cardiac *Fgf23* of 3364 ± 1466 fold after 6 months compared to Ctrl, suggesting an early and stable synthesis over time. Immunofluorescent staining verified a uniform overexpression of Fgf23 in almost all cardiac myocytes of the left ventricle in AAV-Fgf23 mice (Figure 1G).

To investigate the time course of the development of LVH by chronic cardiac Fgf23 overload, we next performed a pilot experiment with each *n* = 3 mice per group investigating heart function up to 6 months after injection of 5 × 10^11^ vg AAV-Fgf23. Analyzed by cardiac MRI, LV mass, LV volumes, and EF were not significantly changed in AAV-Fgf23 mice compared to control, irrespective of the duration of cardiac Fgf23 exposure (Supplementary Table 6). Although not used in the main study because of possible off-target effects in the liver, it is important to note that even the highest AAV-Fgf23 dose of 10^12^ vg did not lead to a classical LVH within 8 weeks (Supplementary Table 7). Taken together, both dose and time-depended pilot studies showed stable expression of *Fgf23* in the heart of AAV-Fgf23 mice from 4 weeks up to 6 months without altering cardiac function.

### Myocardial Gene Transfer of AAV-Fgf23 Causes Cardiac-Specific Synthesis and Secretion of Intact Fgf23

**FIGURE 2:**
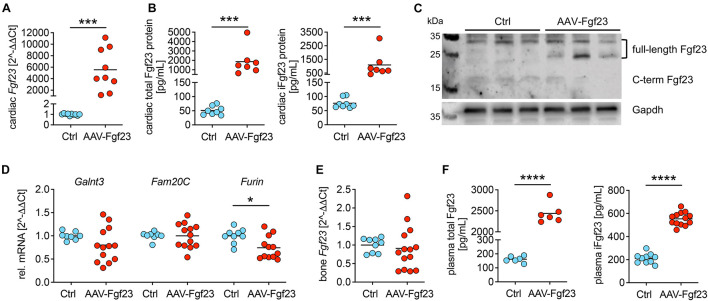
Myocardial gene transfer of AAV-Fgf23 causes cardiac-specific Fgf23 overexpression resulting in the synthesis of intact Fgf23 protein. **(A)** Relative cardiac *Fgf23* mRNA expression increases in AAV-Fgf23 mice compared to Ctrl. **(B)** ELISA-based quantification of cardiac Fgf23 protein in heart tissue lysates shows increased total and intact Fgf23 (iFgf23) protein in AAV-Fgf23 mice compared to Ctrl. **(C)** Representative immunoblotting of Fgf23 in cardiac tissue of three mice each demonstrates increased iFgf23 protein in AAV-Fgf23 mice compared to Ctrl, whereas C-terminal fragments are slightly reduced. Gapdh serves as the loading control. **(D)** Relative mRNA expression of *Galnt3* and *Fam20C* is unchanged in AAV-Fgf23 mice compared to Ctrl, while *Furin* transcription is significantly reduced. **(E)** *Fgf23* mRNA expression in the bone is similar between AAV-Fgf23 and Ctrl mice. **(F)** Plasma C-term and iFgf23 levels are significantly enhanced in AAV-Fgf23 mice compared to Ctrl. Data are given as scatter dot plots with means; *p < 0.05, ***p < 0.001, and ****p < 0.0001 analyzed using unpaired *t*-test or Mann–Whitney test according to Shapiro–Wilk normality test; *n* = 6–14 mice per group.

To investigate the impact of chronic cardiac Fgf23 overload in more detail, we used 5 × 10^11^ vg AAV-Fgf23 for the duration of 4 months. Thereby, we avoid any off-target effects in the liver and impaired cardiac function because of aging. Four months after injecting 5 × 10^11^ vg AAV-Fgf23, cardiac *Fgf23* mRNA levels increased by 5500-fold compared to Ctrl (Figure 2A). Quantification of total and intact cardiac Fgf23 protein demonstrated significant increased concentrations in AAV-Fgf23 mice compared to Ctrl (Figure 2B). Immunoblot revealed a specific increase of ∼26 kDa Fgf23 protein isoform in cardiac tissue lysates of AAV-Fgf23 mice (Figure 2C), indicating mature, full-length iFgf23 protein (Tagliabracci et al., 2014), with no differences in ∼32 kDa iFgf23 isoform. In total, enhanced cardiac iFgf23 protein in immunoblot analysis verified significantly higher iFgf23 protein concentrations in tissue lysates using ELISA-based quantification (Figure 2B). Cardiac mRNA expression of *Galnt3* and *Fam20C* did not show any alterations between AAV-Fgf23 and Ctrl mice (Figure 2D). However, *Furin* was significantly reduced by 25% in AAV-Fgf23 mice, supporting the assumption of immunoblot analysis that cleaved C-terminal Fgf23 fragments seem to be slightly reduced in AAV-Fgf23 mice. Although, administration of AAV-Fgf23 did not enhance *Fgf23* synthesis in bone (Figure 2E), plasma levels of total and iFgf23 increased in AAV-Fgf23 mice compared to Ctrl (Figure 2F), indicating a cardiac origin. Taken together, our data suggest enhanced cardiac-specific overexpression and synthesis of iFgf23 in AAV-Fgf23 mice that is secreted into circulation.

### AAV-Fgf23 Driven Intact Fgf23 Is Biologically Active

**FIGURE 3:**
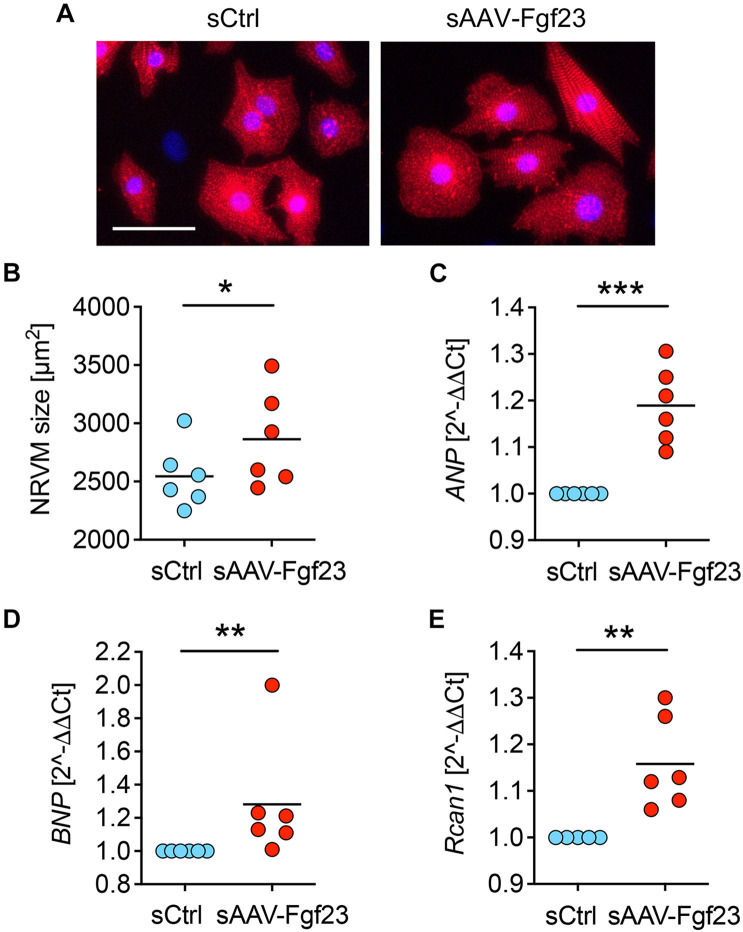
Serum of AAV-Fgf23 mice induces cardiac hypertrophy of NRVM. **(A)** Representative immunofluorescence images of isolated neonatal rat ventricular myocytes (NRVM) stimulated with 2% serum from AAV-Fgf23 (sAAV-Fgf23) or Ctrl (sCtrl) mice for 48 h. Myocytes are labeled with anti-α-actinin (red) and nuclei are counterstained with DAPI (blue) (original magnification ×20; scale bar 50 μm). **(B)** Quantification of the cross-sectional area of NRVM reveals hypertrophic cell growth due to treatment with sAAV-Fgf23 compared to sCtrl (mean of 100 cells per condition and isolation). **(C–E)** Quantitative real-time PCR analysis shows increased expression of *ANP*, *BNP*, and *Rcan1* in sAAV-Fgf23-treated NRVM compared to sCtrl. Data are given as scatter dot plots with means; *p < 0.05, **p < 0.01, ***p < 0.001 analyzed using unpaired *t*-test or Mann–Whitney test according to Shapiro–Wilk normality test; *n* = 6 independent isolations of NRVM.

To investigate the bioactivity of enhanced cardiac-driven circulating iFgf23, we used isolated NRVMs that express all Fgfr isoforms but lack klotho (Faul et al., 2011; Grabner et al., 2015). Cultured NRVM were treated with 2% serum of AAV-Fgf23 (sAAV-Fgf23) or Ctrl (sCtrl) mice for 48 h. Thereby, sAAV-Fgf23 stimulated hypertrophic growth of NRVM (Figures 3A,B) and induced pro-hypertrophic NFAT target genes atrial natriuretic peptide (*ANP*), brain natriuretic peptide (*BNP*), and regulator of calcineurin 1 (*Rcan1*) compared to sCtrl (Figures 3C–E), suggesting that circulating AAV-Fgf23 driven iFgf23 is biologically active.

### Cardiac Fgf23 Excess in Wild-Type Mice Does Not Impair Cardiac Function or Geometry

**FIGURE 4:**
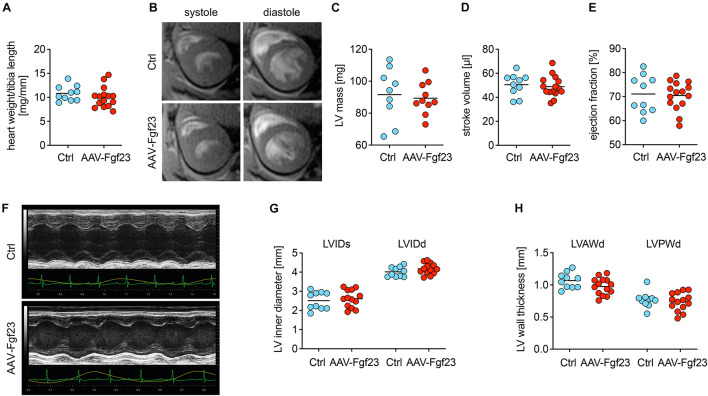
Chronic intra-cardiac Fgf23 synthesis does not impair cardiac function in unchallenged mice. **(A)** The heart weight to tibia length ratio is not increased in AAV-Fgf23 mice compared to Ctrl. **(B)** Representative cross-section MRI images of an AAV-Fgf23 and Ctrl mouse during systole and diastole. **(C–E)** Analyzed by MRI, left ventricular (LV) mass, stroke volume, and ejection fraction show no significant differences in AAV-Fgf23 mice compared to Ctrl. **(F)** Representative PLAX M-mode echocardiography images of an AAV-Fgf23 and Ctrl mouse. **(G,H)** In echocardiography, LV inner diameter during systole and diastole (LVIDs/d) and LV wall thicknesses during diastole (LV anterior wall, LVAW; LV posterior wall, LVPW) are not altered in AAV-Fgf23 mice compared to Ctrl. Data are given as scatter dot plots with means; *n* = 9–15 mice per group.

Despite cardiac Fgf23 overexpression, heart weight to tibia length was not altered in AAV-Fgf23 mice compared to Ctrl (Figure 4A). Analyzed by cardiac MRI, AAV-Fgf23 mice showed no changes in LV mass or heart function, i.e., SV, EF, ESV, and EDV compared to Ctrl (Figures 4B–E and Supplementary Table 8). Although LVAWd was slightly increased in AAV-Fgf23 mice compared to Ctrl, all other LV geometry parameters remained unchanged (Supplementary Table 8). To reaffirm these findings, we performed echocardiography (Figure 4F). In accordance with the MRI results, no significant difference in EF between AAV-Fgf23 and Ctrl mice was observed (Supplementary Table 9). While SV was slightly enhanced in AAV-Fgf23 mice compared to Ctrl, LVIDs, and LVIDd were similar between groups and finally, systolic and diastolic LVAW and LVPW, as well as LV mass and FS were unaffected in AAV-Fgf23 mice (Figures 4G,H and Supplementary Table 9).

### Pathological Cardiac Remodeling Is Not Induced in AAV-Fgf23 Mice

**FIGURE 5:**
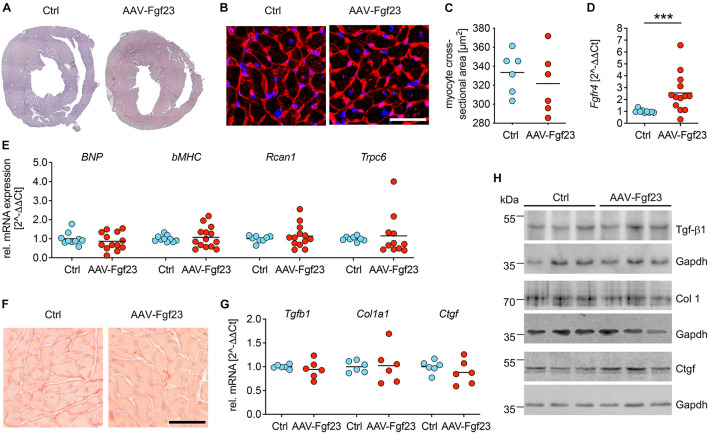
Adeno-associated virus expressing murine Fgf23 mice do not show any signs of pathological LVH. **(A)** Representative cross-sections of AAV-Fgf23 and Ctrl mice stained with HE (original magnification ×10). **(B)** Representative cross-sections of AAV-Fgf23 and Ctrl mice stained and wheat germ agglutinin (WGA) (original magnification ×40; scale bar, 50 μm). **(C)** Quantification of at least 100 individual cardiac myocytes per mouse reveals no size differences between both groups. **(D)** As analyzed by quantitative real-time PCR, cardiac *Fgfr4* mRNA expression is significantly enhanced in AAV-Fgf23 mice compared to Ctrl. **(E)** The pro-hypertrophic NFAT target genes *BNP*, *bMHC*, *Rcan1*, and *Trpc6* are not induced in AAV-Fgf23 mice compared to Ctrl. **(F)** Representative images of picrosirius red-stained mid-chamber free-wall of AAV-Fgf23 and Ctrl mice (original magnification, ×63; scale bar, 50 μm). **(G,H)** The mRNA and protein expression of fibrosis-associated markers Tgfb1, Col1a1, and Ctgf and are not altered in AAV-Fgf23 mice. Gapdh serves as the loading control. Data are given as scatter dot plots with means; ***p < 0.001 analyzed using Mann–Whitney test according to D’Agostino and Pearson’s normality test; *n* = 6–14 mice per group.

Cross-sections of cardiac mid-chamber and cardiac myocyte size, obtained using WGA staining, revealed no hypertrophic cell growth in AAV-Fgf23 compared to Ctrl (Figures 5A–C). Although, expression of *Fgfr4* was significantly increased in AAV-Fgf23 mice compared to Ctrl (Figure 5D), there were no differences in *BNP*, beta-myosin heavy chain (*bMHC*), *Rcan1*, and transient receptor potential cation channel subfamily C member 6 (*Trpc6*) expression (Figure 5E), all of which are NFAT target genes associated with pathological cardiac hypertrophy. Picrosirius red staining revealed no differences of LV fibrosis between both groups (1.6 ± 0.3 vs. 1.7 ± 0.2%, *p* = 0.4179) (Figure 5F). In addition, mRNA expression and protein levels of pro-fibrotic markers transforming growth factor beta 1 (Tgfb1), collagen 1 (Col1a1), and connective tissue growth factor (Ctgf) were not induced in AAV-Fgf23 mice compared to Ctrl (Figures 5G,H). In conclusion, AAV-Fgf23 mice did not show a pathological cardiac phenotype.

### Enhanced Secretion of Cardiac Intact Fgf23 Decreases Renal Transcription of *NaPi2a*, *NaPi2c*, and *Cyp27b1*

**FIGURE 6:**
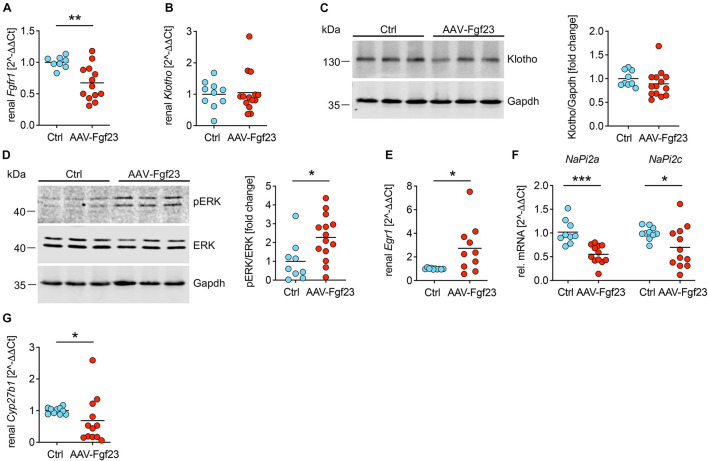
Enhanced secretion of cardiac iFgf23 activates FGFR1/klotho/ERK signaling in the kidney. **(A)** Analyzed by quantitative real-time PCR, renal expression of *Fgfr1* is decreased in AAV-Fgf23 mice compared to Ctrl. **(B)** Renal *Klotho* mRNA levels are equal in both groups. **(C)** Representative immunoblots followed by quantification verify normal klotho protein levels in kidney tissue of AAV-Fgf23 mice compared to Ctrl. Gapdh serves as the loading control. **(D)** Representative immunoblots in kidney tissue followed by quantification show increased phosphorylation of ERK1/2 in AAV-Fgf23 mice compared to Ctrl with no changes of total ERK1/2 protein. Gapdh serves as the loading control. **(E)** Renal mRNA expression of *Egr1* increases in AAV-Fgf23 mice compared to Ctrl, confirming AAV-Fgf23 mediated activation of ERK signaling pathway. **(F)** Renal mRNA expression of *NaPi2a* and *NaPi2c* decreases in AAV-Fgf23 mice compared to Ctrl. **(G)** mRNA expression of *Cyp27b1* is downregulated in AAV-Fgf23 mice compared to Ctrl. Data are given as scatter dot plots with means; *p < 0.05, **p < 0.01, and ***p < 0.001 analyzed using unpaired *t*-test or Mann–Whitney test according to D’Agostino and Pearson’s normality test; *n* = 8–14 mice per group.

To test whether cardiac-derived circulating iFgf23 exerts endocrine function on the kidney, we investigated renal phosphate handling and vitamin D metabolism (Table 1). Although renal mRNA expression of *Fgfr1* was slightly downregulated in AAV-Fgf23 mice compared to Ctrl, no differences in klotho mRNA and protein levels were observed (Figures 6A–C), suggesting physiological soluble klotho (sKL) levels in the circulation. ERK1/2 was phosphorylated in the kidney of AAV-Fgf23 mice and the expression of early growth response 1 (*Egr1*) was up-regulated (Figures 6D,E), indicating Fgf23-dependent activation of renal Fgfr1/klotho/ERK1/2 signaling pathway. AAV-Fgf23 mice showed significantly decreased renal *NaPi2a* and *NaPi2c* mRNA expression (Figure 6F) and phosphate reabsorption compared to Ctrl, although serum phosphate levels were not altered (Table 1). Renal 1α-hydroxylase, encoded by *Cyp27b1*, was significantly suppressed in AAV-Fgf23 mice and associated with higher PTH plasma levels compared to Ctrl (Figure 6G and Table 1), although the latter did not reach the level of statistical significance. Taken together, enhanced circulating iFgf23 in AAV-Fgf23 mice caused down-regulation of sodium-phosphate cotransporters and *Cyp27b1 via* activation of renal Fgfr1/klotho/ERK1/2 signaling, further supporting the hypothesis that cardiac-derived iFgf23 is biologically active.

**TABLE 1:** Kidney function and mineral metabolism in AAV-Fgf23 and control mice.

**Parameter**	**Ctrl**	**AAV-Fgf23**	***p*-Value**
Serum creatinine (mg/dL)	1.42 ± 0.05	1.41 ± 0.06	0.8680
BUN (mg/dL)	20.08 ± 1.00	23.71 ± 1.82	0.1101
Serum phosphate (mg/dL)	8.33 ± 0.72	8.37 ± 0.85	0.9708
Urinary phosphate (mg/dL)	8.66 (2.65; 37.46)	46.28 (23.32; 53.03)	0.0519
TRP (%)	77 ± 10	52 ± 17	0.2148
Serum calcium (mg/dL)	10.52 ± 0.26	10.41 ± 0.50	0.8750
Urinary calcium (mg/dL)	14.88 ± 4.06	17.67 ± 2.25	0.5318
Plasma PTH (pg/mL)	237 ± 18	280 ± 45	0.0543

*Data are presented as mean ± SEM or median (IQR) of each *n* = 5–8 mice per group. BUN, blood urea nitrogen; TRP, tubular reabsorption of phosphate; PTH, parathyroid hormone.*

### Soluble Klotho Prevents Cardiac Intact Fgf23-Induced Cardiac Myocyte Hypertrophy

**FIGURE 7:**
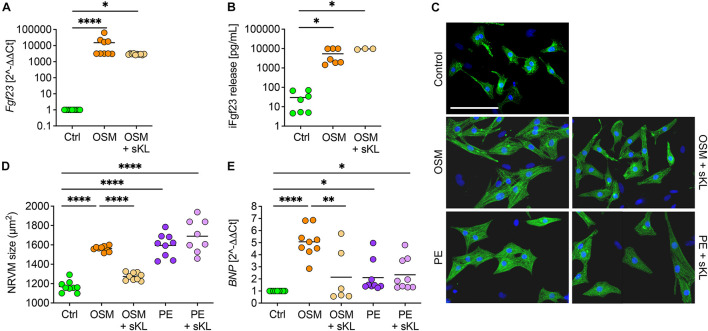
Soluble klotho suppresses cardiac FGF23-induced cardiac hypertrophy *in vitro*. **(A)** Stimulation with oncostatin M (OSM) increases endogenous *Fgf23* mRNA expression in isolated NRVM irrespective of cotreatment with soluble klotho (sKL). **(B)** ELISA-based quantification of intact Fgf23 (iFgf23) in conditioned medium of NRVM reveals enhanced iFgf23 release due to OSM treatment, while sKL costimulation does not alter OSM-mediated high iFgf23 secretion. **(C,D)** Immunofluorescence images of isolated NRVM and quantification of cross-sectional cell area observe hypertrophic growth of OSM stimulated NRVM. Phenylephrine (PE) serves as the positive control for cardiac hypertrophy. Cotreatment with sKL protects from cardiac hypertrophy induced by OSM but not by PE. Myocytes are labeled with anti-α-actinin (green) and nuclei are counterstained with DAPI (blue) (original magnification ×20; scale bar 100 μm). **(E)** Quantitative real-time PCR analysis shows increased expression of *BNP* in OSM and PE-treated NRVM compared to Ctrl that is only blocked by sKL in the OSM group. Data are given as scatter dot plots with means; ^∗^*p* < 0.05, ^∗∗^*p* < 0.01, and ^*⁣*⁣**^p < 0.0001 analyzed using one-way ANOVA or Kruskal–Wallis test followed by Sidak’s or Dunn’s multiple comparisons *post hoc* tests, respectively, according to Shapiro–Wilk normality test; *n* = 6–9 independent isolations of NRVM; conditioned medium was used from *n* = 3–7 independent NRVM isolations.

Although acting on the kidney, cardiac Fgf23 overexpression in healthy mice did not result in a pathological cardiac phenotype, raising the question of how AAV-Fgf23 mice are protected from LVH. Therefore, the physiological klotho synthesis in AAV-Fgf23 mice (Figures 6B,C) might be a protective factor. To address if sKL prevents cardiac myocyte hypertrophy in the setting of increased endogenous cardiac Fgf23 production, we used klotho deficient NRVM *in vitro*. To mimic cardiac-specific Fgf23 overexpression, such as in our AAV-Fgf23 mouse, we used OSM, a cytokine known to induce endogenous cardiac *Fgf23* synthesis in the failing heart (Richter et al., 2012, 2015). After treatment with OSM, *Fgf23* was strongly up-regulated in NRVM compared to Ctrl (Figure 7A) and levels of iFgf23 were significantly higher in the conditioned medium of OSM-treated NRVMs (Figure 7B). In klotho-free culture conditions, the cell size of OSM-treated NRVMs was significantly larger compared to Ctrl and similar to that of PE-stimulated NRVMs as a positive control (Figures 7C,D). Moreover, *BNP* mRNA expression was up-regulated in OSM and PE-stimulated NRVMs compared to Ctrl (Figure 7E). Although the addition of sKL did neither reduce endogenous Fgf23 synthesis nor its release, it inhibited the OSM-induced increase of NRVM cell size and *BNP* expression. Notably, the PE-induced hypertrophic growth of NRVM and pro-hypertrophic *BNP* expression remained unaffected by sKL. Thus, our data suggest an antagonization of cardiac Fgf23 by sKL that may have protected AAV-Fgf23 mice from LVH.

## Discussion

Fibroblast growth factor 23 is considered to be a major contributor to the development of LVH, heart failure, atrial fibrillation, and increased cardiovascular and all-cause mortality in CKD (Faul et al., 2011; Kendrick et al., 2011; Scialla et al., 2014; Mehta et al., 2016) and non-CKD (Ix et al., 2012; Kestenbaum et al., 2014; Lutsey et al., 2014) populations. However, it is still intensively discussed whether elevated FGF23 is cardiotoxic *per se* (Marthi et al., 2018; Pastor-Arroyo et al., 2018; Zhou et al., 2018; Bao et al., 2020). All previously reported animal models developing LVH in the presence of elevated circulating Fgf23 levels due to experimental uremia, genetic deletion of klotho, high phosphate diet, or injection of recombinant FGF23 protein display a variety of systemic changes that are all proven contributors to cardiac injury, making it difficult to elucidate the causative role of FGF23 for LVH in these studies (Faul et al., 2011; Andrukhova et al., 2014; Grabner et al., 2015; Hu et al., 2015; Yang et al., 2015; Leifheit-Nestler et al., 2017). Here, we generated a mouse model with high intra-cardiac synthesis of Fgf23 using AAV-mediated myocardial gene transfer to elucidate the causative role of high FGF23 for LVH without any further pathologies. AAV-Fgf23 injection resulted in elevated biologically active cardiac iFgf23 synthesis. Although AAV-Fgf23 mice showed unchanged *Fgf23* mRNA expression in the bone, plasma total and iFgf23 levels were increased compared to Ctrl, suggesting a cardiac origin. Interestingly, Fgf23 plasma concentrations were at least as high as circulating Fgf23 levels reported to cause LVH and fibrosis in mice after injection of recombinant FGF23 protein or feeding a high phosphate diet (Faul et al., 2011; Grabner et al., 2017), but AAV-Fgf23 mice did not show a pathological cardiac phenotype.

Of course, *in vitro* studies cannot capture the complexity of whole organ systems. However, isolated NRVM treated with sAAV-Fgf23 clearly showed hypertrophic cell growth and induction of classical pro-hypertrophic NFAT target genes *in vitro*. Thus, secreted cardiac iFgf23 in AAV-Fgf23 mice is to be considered biologically active. This assumption is further supported by fact that AAV-Fgf23 mice showed phosphorylation of ERK1/2 in the kidney and increased renal expression of *Egr1* with concomitant reduced transcription of *NaPi2a* and *NaPi2c* as well as *Cyp27b1*, all of which are well-characterized, FGF23-mediated physiological signaling pathways in the kidney (Urakawa et al., 2006; Gattineni et al., 2009). Nevertheless, the absence of a pathological cardiac phenotype raises the question of why AAV-Fgf23 mice are protected from LVH.

First evidence that FGF23 may promote LVH came from clinical association studies in CKD patients (Gutierrez et al., 2008, 2009; Leifheit-Nestler et al., 2016), which was verified in different rodent models of experimental uremia (Faul et al., 2011; Leifheit-Nestler et al., 2017; Czaya et al., 2019). Recently, it was postulated that elevated circulating FGF23 does not increase cardiovascular disease (CVD) risk without CKD (Pastor-Arroyo et al., 2018). AAV-Fgf23 mice in the present study showed normal kidney function and thus, this might explain why high circulating and cardiac Fgf23 levels without the uremic milieu did not promote pathological LVH. However, to date, FGF23 has not been shown to be causally responsible for LVH in CKD. In contrast, a recent study suggests that high FGF23 in CKD does not cause CVD but is rather a consequence of it (Zhou et al., 2018).

Chronic kidney disease is among others characterized by a disturbed FGF23-associated mineral metabolism and studies support the concept that concomitant hyperphosphatemia may promote FGF23’s cardiac toxicity. In patients with end-stage kidney disease (ESKD) developing LVH, high serum phosphate levels were associated with enhanced expression of cardiac FGF23 that associated with the presence of LVH (Leifheit-Nestler et al., 2016). In comparative *in vivo* studies, LVH was only present in mice with high intra-cardiac Fgf23 synthesis and hyperphosphatemia and not in those with normal serum phosphate concentrations (Leifheit-Nestler et al., 2018b). By investigating several uremic toxins, high phosphate had the strongest ability to impair fatty acid oxidation in H9c2 rat cardiomyoblast cells and NRVM contributing to cardiac hypertrophy *in vitro* (Huang et al., 2020). The authors further postulated that high phosphate contributes to LVH in experimental CKD by altering myocardial energy metabolism. AAV-Fgf23 mice showed normal serum phosphate levels and may therefore be protected against pathological cardiac remodeling. This assumption is consistent with studies in murine models of X-linked hypophosphatemia that could not detect a pathological cardiac phenotype despite markedly elevated Fgf23 (Leifheit-Nestler et al., 2018b; Liu et al., 2018; Pastor-Arroyo et al., 2018). In the same regard, cardiac hypertrophy due to elevated phosphate and Fgf23 was reversed by lowering dietary phosphate intake *in vivo* (Grabner et al., 2017), supporting the hypothesis that cooperative interaction of FGF23 and high phosphate needs to be present for the development of LVH in CKD and non-CKD. On a molecular level, phosphate was recently shown to induce the expression of *Galnt3* in osteoblastic UMR106 cells catalyzing Fgf23’s O-glycosylation on threonine 178 and thereby protects Fgf23 from cleavage (Takashi et al., 2019). In CKD, serum phosphate concentration rises and the magnitude of increased bone FGF23 production and failure of adequate coupling of FGF23 cleavage results in increased circulating levels of biologically active iFGF23 (Wolf and White, 2014). It may well be that chronically high phosphate stabilizes its phosphaturic hormone also in cardiac myocytes locally thereby contributing to the development of FGF23-mediated LVH. Of course, this has to be confirmed in further studies.

Despite impaired phosphate homeostasis, declining renal klotho synthesis leading to sKL deficiency is discussed as a further pathology in CKD. Experimental studies have shown that FGF23 induces LVH *via* FGFR4-induced activation of calcineurin/NFAT signaling independent of klotho, because cardiac myocytes do not express klotho (Faul et al., 2011; Grabner et al., 2015). Likewise, klotho hypomorphic mice develop severe cardiac hypertrophy and fibrosis with activation of the calcineurin/NFAT and TGF-β signaling pathways (Faul et al., 2011; Leifheit-Nestler et al., 2018b), and heterozygous klotho-deficient mice show decreased EF, SV, cardiac output, and impaired cardiac geometry (Hu et al., 2015). Although klotho is not expressed in the myocardium, sKL most likely originating from the circulation was detected in the human heart and cardiac klotho protein content was negatively associated with the development of pathological LVH in ESKD patients (Leifheit-Nestler et al., 2016, 2018a). Thus, normal renal klotho synthesis in AAV-Fgf23 mice indicates physiological sKL levels in the circulation that may have protected unchallenged AAV-Fgf23 mice from pathological cardiac remodeling. Cardioprotective properties of klotho have previously been attributed to its suppressive effects on reactive oxygen species, reducing the severity of indoxyl sulfate-induced hypertrophy, and inhibition of Trpc6 as demonstrated in an animal model of stress-induced cardiac hypertrophy (Xie et al., 2012; Yang et al., 2015; Zhu et al., 2017). *Trpc6* was normally transcribed in AAV-Fgf23 mice, suggesting an inhibitory effect of normal sKL in the present study.

A recent study showed that sKL acts directly as a scaffold protein for FGF23 and FGFRs promoting FGF23/FGFR1c-mediated signaling, and may thereby induce a switch in the FGF23 signaling pathway from FGFR4 to FGFR1c that is discussed to be another potential cardioprotective mechanism of klotho (Chen et al., 2018; Richter and Faul, 2018). The assumption that klotho acts as a cardioprotective reagent is also supported by our *in vitro* data showing prevention of cardiac Fgf23-induced cardiac hypertrophy by sKL in NRVMs without altering high intra-cardiac Fgf23 expression and secretion. Our data further indicate that enhanced cardiac-specific FGF23 synthesis has the potential to directly act on the heart inducing pathological cardiac changes *via* paracrine mechanisms.

Faul et al. (2011) presented evidence that FGF23 can trigger cardiac hypertrophy in the context of klotho deficiency *in vitro* and *in vivo*, which was later confirmed in other studies (Leifheit-Nestler et al., 2016, 2018a; Böckmann et al., 2019; Schön et al., 2021). All of these studies are in line with the present *in vitro* data. However, Faul et al. (2011) additionally showed that the intramyocardial or intravenous injection of recombinant Fgf23 protein in wild-type mice caused LVH as well. Methodological differences between the two studies may at least partly explain the conflicting results. Faul et al. (2011) enhanced circulating FGF23 and here we overexpressed endogenous cardiac FGF23. In addition, Faul et al. (2011) used a cleavage resistant recombinant Fgf23 protein and we administered a murine wild-type Fgf23 cDNA cloned into an AAV construct so that Fgf23 protein can still be posttranslational modified. To date, it is controversially discussed whether high FGF23 causes LVH in healthy individuals or increased FGF23 is just a result of cardiac hypertrophy. To the best of our knowledge, we are the first to investigate paracrine effects of high endogenous cardiac Fgf23 synthesis under normal conditions.

Whether or not hyperphosphatemia and/or reduced klotho levels are the major driver for the cardiac toxicity of high FGF23 in CKD has to be confirmed in further studies. Nonetheless, our data suggest that high endogenous cardiac FGF23 synthesis has the potential to directly act on the heart inducing pathological cardiac changes *via* paracrine mechanisms that are blocked by sKL protecting unchallenged AAV-Fgf23 mice from LVH.

## Data Availability Statement

The original contributions presented in the study are included in the article/Supplementary Material, further inquiries can be directed to the corresponding author.

## Ethics Statement

The animal study was reviewed and approved by Niedersächsisches Landesamt für Verbraucherschutz und Lebensmittelsicherheit (LAVES).

## Author Contributions

ML-N and DH designed the study. ML-N, MAW, BR, CP, FE, IB, IV, and AG carried out the experiments and analyzed the data. SSH produced the AAVs. KZ performed and AF analyzed the echocardiography. TT discussed the echocardiographic results. OJM designed and provided the AAVs. ML-N designed the figures. ML-N, MAW, BR, and DH drafted and revised the manuscript. All authors approved the final version of the manuscript.

## Conflict of Interest

TT filed and licensed patents in the field of non-coding RNAs and is the founder and shareholder of Cardior Pharmaceuticals GmbH. The remaining authors declare that the research was conducted in the absence of any commercial or financial relationships that could be construed as a potential conflict of interest.

## Publisher’s Note

All claims expressed in this article are solely those of the authors and do not necessarily represent those of their affiliated organizations, or those of the publisher, the editors and the reviewers. Any product that may be evaluated in this article, or claim that may be made by its manufacturer, is not guaranteed or endorsed by the publisher.
