# Supplementary material for: Cardiac Fibroblast Growth Factor 23 Excess Does Not Induce Left Ventricular Hypertrophy in Healthy Mice
Source: Front Cell Dev Biol. 2021 Oct 28;9:745892. doi: 10.3389/fcell.2021.745892 (PMC8581397; doi:10.3389/fcell.2021.745892)
Supplement: Supplementary file 1 [file Data_Sheet_1.docx]

Supplementary Material

**Supplementary Table 1.** Murine qRT-PCR primer sequences.

| Gene | Orientation | Sequence (5`-3`) |
| --- | --- | --- |
| bMHC | forward | AGG CAA GGC AAA GAA AGG CTC AT |
|  | reverse | GCG TGG AGC GCA AGT TTG TCA TA |
| BNP | forward | GAG GTC ACT CCT ATC CTC TGG |
|  | reverse | GCC ATT TCC TCC GAC TTT TCT C |
| Col1a1 | forward | CCG CTG GTC AAG ATG GTC |
|  | reverse | CCT CGC TCT CCA GCC TTT |
| Ctgf | forward | CTG CCT GGG AAA TGC TGC GAG GA |
|  | reverse | GTT GGG TCT TGG GCC AAA TGT |
| Egr1 | forward | GAC AGC AGT CCC ATT TAC TC |
|  | reverse | ATC TTG GTA TGC CTC TTG CG |
| Fam20C | forward | CGC GGG ATA AGA AGC TAT GG |
|  | reverse | CGC GGG ATA AGA AGC TAT GG |
| Fgfr1 | forward | TGC CAG CTG CCA AGA CGG TG |
|  | reverse | AAG GAT GGG CCG GTG AGG GG |
| Fgfr4 | forward | GGC TAT GCT GTG GCC GCA CT |
|  | reverse | GGT CTG AGG GCA CCA CGC TC |
| Fgf23 |  | QuantiTect Primer Assay, QT01166557 |
| Furin | forward | AGC CAA GAG GGA CGT GTA TC |
|  | reverse | CTC GCT GAG TGA CAC CAG AC |
| Galnt3 | forward | ACA CTA TTT ACC CGG AAG CG |
|  | reverse | AGC TCC TTC TGG ATG TTG TG |
| Gapdh | forward | TAT GTC GTG GAG TCT ACT GG |
|  | reverse | AGT GAT GGA TGG ACT GTG G |
| Klotho | forward | ACT ACG TTC AAG TGG ACA CTA C |
|  | reverse | TCA ACA CAG TAA GGT TTT CTC T |
| NaPi2a | forward | AGA CAC AAC AGA GGC TTC |
|  | reverse | CCA AAG GAG GAT AAG ACA AG |
| NaPi2c | forward | CAT CTT CAA CTG GCT CAC |
|  | reverse | GGT TAT CAC ACT GCT ATC C |
| Rcan1 | forward | GCT TGA CTG AGA GAG CGA GTC |
|  | reverse | CCA CAC AAG CAA TCA GGG AGC |
| Rsp18 | forward | TTC TGG CCA ACG GTC TAG ACA AC |
|  | reverse | CCA GTG GTC TTG GTG TGC TGA |
| Tgfb1 | forward | TTG CTT CAG CTC CAC AGA GA |
|  | reverse | TGG TTG TAG AGG CAA AC |
| Trpc6 | forward | CTA CAT TGG CGC AAA ACA GA |
|  | reverse | AGA CCA AAG ATA GCC CAG AAC A |

**Supplementary Table 2.** Rat primer sequences for qRT-PCR.

| Gene | Orientation | Sequence (5`-3`) |
| --- | --- | --- |
| ANP | forward | AAA TCC CGT ATA CAG TGC GG |
|  | reverse | GGA GGC ATG ACC TCA TCT TC |
| BNP | forward | CCA GAA CAA TCC ACG ATG C |
|  | reverse | TCG AAG TCT CTC CTG GAT CC |
| Gapdh | forward | ACT CCA CGA CAT ACT CAG CAC |
|  | reverse | CAT CAA CAC CCC TTC ATT |
| Rcan1 | forward | CTC ACA CAC GTG GAC CAC CA |
|  | reverse | CGC CCA ATC CAG ACA AAC AG |

**Supplementary Table 3.** Murine primer sequences for semi-quantitative PCR.

| Gene | Orientation | Sequence (5`-3`) |
| --- | --- | --- |
| AAV vector | forward | GCC TGT ATC CTG CTC TCG C |
|  | reverse | ATG TTT CAG GTT CAG GGG GAG |

**Supplementary Table 4.** Primary antibodies for immunoblotting.

| Antibody | Species | Buffer | Dilution | Manufacturer |
| --- | --- | --- | --- | --- |
| Col1 | Rabbit | 5% milk + TBST | 1:1,000 | Abcam |
| Ctgf | Rabbit | 5% BSA + TBST | 1:1,000 | Abcam |
| ERK1/2 | Mouse | 5% BSA + TBST | 1:1,000 | Cell Signaling |
| pERK1/2 | Rabbit | 5% BSA + TBST | 1:500 | Cell Signaling |
| FGF23 (225-244) | Goat | 5% milk + TBST | 1:500 | Immutopics |
| Gapdh | Rabbit | 5% milk + TBST / 5% BSA + TBST | 1:1,000/  1:10,000 | Cell Signaling |
| Klotho | Goat | 5% milk + TBST | 1:1,000 | R&D Systems |
| Tgfb1 | Rabbit | 5% milk + TBST | 1:500 | Abcam |

**Supplementary Table 5.** Secondary antibodies for immunoblotting.

| Antibody | Species | Buffer | Dilution | Manufacturer |
| --- | --- | --- | --- | --- |
| Anti-mouse IRDye | Mouse | 5% BSA + TBST | 1:5,000 | LICOR |
| Anti-rabbit IRDye | Goat | 5% BSA + TBST | 1:10,000 | LICOR |
| Anti-goat HRP | Donkey | 5% Milk + TBST | 1:2,000 | R&D Systems |
| Anti-rabbit HRP | Mouse | 5% Milk + TBST | 1:1,000 or  1:2,000 | Santa Cruz Biotechnology |

**Supplementary Table 6.** Quantification of cardiac magnetic resonance imaging three and six months after 5x10^11^ vg AAV-Fgf23 injection compared to Ctrl.

| **Parameter** | **3 months** | | **6 months** | |
| --- | --- | --- | --- | --- |
|  | **Ctrl**  (*n*=3) | **AAV-Fgf23**  (*n*=3) | **Ctrl**  (*n*=3) | **AAV-Fgf23**  (*n*=3) |
| LV mass [mg] | 71.8±14.9 | 73.2±5.2 | 70.7±3.6 | 84.9±5.4 |
| SV [µL] | 30.1±4.2 | 32.7±3.5 | 29.9±0.01 | 35.4±4.4 |
| ESV [µL] | 27.5±0.5 | 22.7±9.0 | 18.5±3.5 | 25.0±0.7 |
| EDV [µL] | 54.8±10.0 | 55.4±6.0 | 48.4±3.5 | 60.4±3.7 |
| EF [%] | 56.3±6.0 | 59.8±11.5 | 61.9±4.5 | 58.5±3.7 |

Values are presented as mean±SD and *p* values calculated using unpaired *t*-test. LV mass, left ventricular mass; SV, stroke volume; ESV, end-systolic volume; EDV, end-diastolic volume; EF, ejection fraction.

**Supplementary Table 7.** Quantification of cardiac magnetic resonance imaging four and eight weeks after 10^12^ vg AAV-Fgf23 injection compared to Ctrl.

| **Parameter** | **4 weeks** | | **8 weeks** | |
| --- | --- | --- | --- | --- |
|  | **Ctrl**  (*n*=3) | **AAV-Fgf23**  (*n*=3) | **Ctrl**  (*n*=3) | **AAV-Fgf23**  (*n*=3) |
| LV mass [mg] | 62.1±9.0 | 71.0±7.8 | 63.7±3.5 | 63.0±4.8 |
| SV [µL] | 21.0±1.0 | 24.2±3.1 | 22.3±2.8 | 23.8±1.4 |
| ESV [µL] | 11.5±0.9 | 11.3±2.3 | 11.4±1.7 | 12.4±1.6 |
| EDV [µL] | 32.6±0.2 | 35.5±4.3 | 33.7±1.3 | 36.2±2.0 |
| LVAWs [mm] | 1.15±0.16 | 1.03±0.45 | 0.96±0.01 | 1.19±0.30 |
| LVAWd [mm] | 0.71±0.28 | 0.66±0.23 | 0.70±0.12 | 0.66±0.09 |
| LVPWs [mm] | 1.74±0.22 | 1.65±0.22 | 1.61±0.28 | 1.32±0.08 |
| LVPWd [mm] | 1.30±0.28 | 1.33±0.11 | 0.97±0.23 | 0.75±0.28 |
| LVIDs [mm] | 1.92±0.30 | 2.12±0.47 | 1.53±0.29 | 1.56±0.11 |
| LVIDd [mm] | 2.92±0.29 | 2.68±0.12 | 2.64±0.23 | 3.01±0.47 |
| EF [%] | 64.6±2.9 | 68.2±4.3 | 68.1±5.9 | 65.8±3.4 |

Values are presented as mean±SD and *p* values calculated using unpaired *t*-test. LV mass, left ventricular mass; SV, stroke volume; ESV, end-systolic volume; EDV, end-diastolic volume; LVAWs/d, left ventricular anterior wall thickness during systole/; LVPWs/d, left ventricular posterior wall thickness during systole/diastole; LVIDs/d, left ventricular inner diameter during systole/diastole; EF, ejection fraction.

**Supplementary Table 8.** Quantification of cardiac magnetic resonance imaging four months after 5x10^11^ vg AAV-Fgf23 injection compared to Ctrl.

| **Parameter** | **Ctrl**  (*n*=8-10) | **AAV-Fgf23**  (*n*=14-15) | ***p* value** |
| --- | --- | --- | --- |
| LV mass [mg] | 91.6±16.8 | 89.3±9.9 | 0.7192 |
| SV [µL] | 50.5±9.1 | 48.9±8.7 | 0.6604 |
| ESV [µL] | 20.9±7.9 | 20.5±5.1 | 0.8643 |
| EDV [µL] | 71.5±13.9 | 69.4±10.9 | 0.6821 |
| LVAWs [mm] | 1.32±0.16 | 1.41±0.18 | 0.2794 |
| LVAWd [mm] | 1.06 [1.02; 1.09] | 1.16 [1.08; 1.22] | 0.0291 |
| LVPWs [mm] | 2.08±0.44 | 2.16±0.27 | 0.5596 |
| LVPWd [mm] | 1.17±0.14 | 1.19±0.20 | 0.7776 |
| LVIDs [mm] | 2.20 [1.88; 2.54] | 1.96 [1.81; 2.21] | 0.3188 |
| LVIDd [mm] | 4.07 [3.93; 4.28] | 3.96 [3.88; 4.22] | 0.3296 |
| EF [%] | 71.1±7.8 | 70.5±5.9 | 0.8213 |
| FS [%] | 45.5 [41.7; 51.2] | 50.7 [44.6; 55.7] | 0.3407 |

Values are presented as mean±SD or median [IQR] and *p* values calculated using unpaired *t*-test or Mann-Whitney test, respectively. LV mass, left ventricular mass; SV, stroke volume; ESV, end-systolic volume; EDV, end-diastolic volume; LVAWs/d, left ventricular anterior wall thickness during systole/; LVPWs/d, left ventricular posterior wall thickness during systole/diastole; LVIDs/d, left ventricular inner diameter during systole/diastole; EF, ejection fraction; FS, fractional shortening.

**Supplementary Table 9.** Quantification of echocardiography four months after 5x10^11^ vg AAV-Fgf23 injection compared to Ctrl.

| **Parameter** | **Ctrl**  (*n*=10) | **AAV-Fgf23**  (*n*=14) | ***p* value** |
| --- | --- | --- | --- |
| LV mass [mg] | 113.1±13.8 | 110.1±19.3 | 0.6809 |
| SV [µL] | 45.9±2.6 | 51.7±9.0 | 0.1251 |
| ESV [µL] | 24.1±10.5 | 26.5±10.8 | 0.5989 |
| EDV [µL] | 70.0±11.7 | 77.1±12.6 | 0.1765 |
| LVAWs [mm] | 1.47 [1.35; 1.58] | 1.39 [1.12; 1.53] | 0.1541 |
| LVAWd [mm] | 1.07±0.12 | 0.98±0.13 | 0.0900 |
| LVPWs [mm] | 1.03 [0.91; 1.15] | 1.12 [0.90; 1.44] | 0.7088 |
| LVPWd [mm] | 0.77±0.12 | 0.74±0.14 | 0.5901 |
| LVIDs [mm] | 2.52±0.48 | 2.63±0.45 | 0.5838 |
| LVIDd [mm] | 3.99±0.28 | 4.15±0.29 | 0.1755 |
| EF [%] | 67.0±10.1 | 66.3±11.3 | 0.8858 |
| FS [%] | 37.3±8.1 | 37.0±8.4 | 0.9290 |

Values are presented as mean±SD or median [IQR] and *p* values calculated using unpaired *t*-test or Mann-Whitney test, respectively. LV mass, left ventricular mass; SV, stroke volume; ESV, end-systolic volume; EDV, end-diastolic volume; LVAWs/d, left ventricular anterior wall thickness during systole/diastole; LVPWs/d, left ventricular posterior wall thickness during systole/diastole; LVIDs/d, left ventricular inner diameter during systole/diastole; EF, ejection fraction; FS, fractional shortening.
